# Hunger Whilst “*In Utero*” Programming Adult Osteoporosis

**DOI:** 10.5041/RMMJ.10138

**Published:** 2014-01-21

**Authors:** George M. Weisz, William R. Albury

**Affiliations:** 1 Department of Orthopaedics, Sydney Wolper Jewish Hospital, School of Humanities, University of New England, Armidale, NSW, Australia, and School of Humanities (Program in History of Medicine), University of NSW, Sydney, Australia and; 2 School of Humanities, University of New England, Armidale, NSW, Australia

**Keywords:** Ghettos, glucose, lipid, mineral metabolism, starvation

## Abstract

The fetal “programming of adult diseases” has been previously reviewed. The descriptions were comprehensive, dealing with the effects of nutritional deprivation on the development of adult metabolic and cardiovascular diseases. During the past decade, research into this “programming” also expanded to the development of osteoporosis. The present review deals with the imbalance of bone mineral metabolism, “programmed” by maternal/fetal/infantile nutritional deprivation, and is illustrated with a family history from the Budapest Ghetto.

## INTRODUCTION

Gestational, immediately postnatal, and early infantile nutrition has been recognized as an influential factor in programming adult metabolic diseases. This concept was previously reviewed by Ophir and Oettinger,^1^ with a detailed epidemiological review offered by Ben-Shlomo and Kuh,^2^ and a further updated review was offered by Hazani and Shasha.^3^ These reviews dealt with fetal and infantile exposure to insults and their effect on the development of adult illnesses. The effects of external stimuli manifest as changes appearing in a “critical period” of gestation—such as the teratogenic effect of thalidomide on limb development, the effect of rubella on cardiac development, or the effect of radiation on brain cell formation. Another example is the effect of reduced folic acid on the development of the vertebral canal (spina bifida, with or without the exteriorized meningocele).

Other changes also appear as an accumulating effect in a “sensitive period,” with pathology surfacing later in adult life, such as the carcinogenic effect of diethylstilbestrol (DES) during gestation, predisposing women to vaginal and uterine carcinoma as teenagers or adults.

Another important past-genomic influence was found to be gestational nutrition.

## GESTATIONAL DEVELOPMENT OF THE MUSCULOSKELETAL SYSTEM

A relatively less studied embryonic aspect is the development of musculoskeletal pathology (e.g. adipose tissue, sarcopenia, osteopenia).^4^,^5^ The theory of fetal programming of body composition and musculoskeletal development has been previously well defined.^4^ Maternal and fetal malnutrition were found to be programmers of muscle, bone, and adipose tissue development and are trimester-sensitive.^5^ In normal muscle development the primary muscle fibers are produced in the first trimester (6–8 weeks) and further multiplied by the secondary fibers in the later trimester (8–18 weeks). It was documented, both experimentally and epidemiologically, that fetal nutritional deprivation can lead to sarcopenia in adulthood.^5^ Fat tissue development occurs mainly in the third trimester (30 weeks). Malnutrition predisposes infants to low birth weight, compensated for with a “catch-up” metabolism. Both experimental and epidemiological studies suggest that fetal nutritional deprivation can program obesity in adulthood.

Bone development starts with osteoblastic invasion of the embryonic cartilaginous skeleton early in the first trimester (5 weeks), then continues during gestation and also in the postnatal period. It was documented that fetal nutritional deprivation, hypovitaminosis D, and low calcium intake are all factors which can program osteopenia in adults.^6^

## THE EFFECT OF EARLY-LIFE STARVATION ON THE DEVELOPMENT OF ADULT DISEASES

During WWII, starvation was used by the German authorities as a weapon of submission or punishment in the siege of Leningrad and in the Netherlands. Starvation also occurred on the Channel Islands when their food supply was cut off by the Normandy invasion.

*The Leningrad siege*: The German army surrounded the city of 2.9 million (0.5 million children) between September 1941 and January 1944, resulting in 630,000 deaths. Many years later, studies were conducted on children born to mothers with sustenance of between 300–800 daily calories during their pregnancy. There was a clear relationship between birth size and obesity, with metabolic diseases emerging in infancy and adolescence, and cardiac disease emerging in adulthood.^7^

*The Dutch embargo*: During November 1944, as a reprisal for a railway strike, a severe food embargo was instituted over the Western Netherlands. The caloric supply was gradually reduced to 1000, then to 800, and by April 1945 to 400 calories a day. The registry recorded some 18,000 deaths directly (and several thousands indirectly) related to famine. Later records indicated that individuals with a history of small size and low birth weight were exposed to metabolic aberration (glucose and lipid), to obesity, and to an increased mortality rate by age 50; all this in a cohort compared to the non-affected Eastern Netherlands population.^8^ Also identified were changes in reproductive functions, early menopause, and increased incidence of breast and colon malignancy.^9^–^11^

*The famine in five Channel Islands* off Normandy, involving 60,000 islanders: In 1940 these islands were demilitarized by the British government, with a fifth of the population being evacuated (children, women, and Jews). A five-year-long German repression followed. The co-operation of locals with the occupiers was recorded, as was the betrayal of a few hiding Jews. The invasion of Normandy in 1944 by-passed the islands. The food supply from the continent was cut, and by the end of 1944/early 1945 it became critical. The health consequences were studied only in those born before the war.^12^ Infants exposed to sub-nutrition in 1944–45 were found with increased cardiovascular morbidity, delays in puberty, and an increase in breast cancer (although statistically non-significant). This population was compared with the cohort evacuated to England.^13^

The mechanism of “*in utero* programming” of adult illnesses was proposed by Lucas in 1990. It reads as follows: “an early stimulus or insult, operating at a critical or sensitive period results in permanent or long term changes in the structure or function of the organism.”^4^ This theory was researched in different geographical and environmental conditions and was also confirmed experimentally.^7^,^14^–^16^

Lucas applied his concept of pre- and postnatal nutritional influence to the development of bone mineralization.^17^ It was presented as “programming” future osteopenia, and the risk of fractures was more definitively established in the twenty-first century.^18^,^19^ In the presence of sub-nutrition, the mechanism of rapid growth in the second gestational term requires adaptation, namely a slowed down osteoblastic division. It was established that the earlier in life the malnutrition occurred, the greater the likelihood of permanent effects on bone demineralization. This concept was further promoted by describing the relationship between maternal diet, birth weight, and vitamin D receptor genotype alteration, all as a programmer of osteopenia. The predictive value of growth hormone on bone density in elderly women was also established.^20^–^22^

A recent study in Australia, the third largest country with Holocaust survivors, attracted attention to the topic and discussed the specific needs for geriatric management.^23^ The authors have also reviewed metabolic details in a group of survivors in Australia.^24^

*Example of one family of survivors with four siblings*: The immediate effects of starvation on bone metabolism and fractures were established in 1941–42 in a detailed study in the Warsaw Ghetto. Fractures in children were found not to heal, making surgical treatment inexpedient. Autopsy findings in adults detected severe cortical demineralization, as well as matrix decomposition.^25^ To illustrate the long-term effects of early life starvation, the metabolic details of three siblings, survivors of the Budapest Ghetto, and one control within the same family are used. The fracture risk in our small group of survivors is indicative of moderate to severe risk in 5- and 10-year predictions (Table 1).

**Table 1.** Metabolic changes in late adulthood and the calculated fracture risks (for privacy, only Hebrew names are used).

Name	Age While in Ghetto	Duration of Malnutrition	Osteoporosis	Any Fracture Risk (%): 5y / 10y	Hip Fracture Risk (%):5y / 10y
Moshe	4	12 months	+	6.4 / 1.8	1.1 / 2.1
Hannah	1.5	12 months	+	5.0 / 10.3	0.6 / 1.2
Malaach		*In utero*, last trimester till 6 months of postnatal age	++	4.0 / 8.5	0.6 / 1.2
Shoshana	Born 1947		No	2.0 / 4.2	0.1 / 0.2

The risk of fractures within 5 and 10 years in this family were all calculated from the results obtained with dual-energy X-ray absorptiometry (DEXA) scanners, and all were compared with age- and gender-general values.

The Garvan nomogram used in this brief study is an individual risk assessment based on clinical parameters combined with, rather than based only on, bone mineral density (BMD) and/or T-score values [www.Garvan.org.au/bone-fracture-risk/].

The predicted risk and the prognostic values suggest, as expected, that the more intense the osteoporosis, the higher the fracture incidence will be.

Impression: Nutritional deprivation during pregnancy or in the postnatal period can have a “programming” effect on the development of adult glucose and lipid metabolism, a mechanism also detected in the development of osteoporosis. The current opinion in the literature is further enhanced by examples of survivors of the Holocaust.

This preliminary review intends only to raise awareness; it has insufficient statistical data to support firm scientific conclusions. A rigorous epidemiological study should perhaps be undertaken by those with access to a larger community of child survivors.
